# Association of SMAD7 rs12953717 Polymorphism with Cancer: A Meta-Analysis

**DOI:** 10.1371/journal.pone.0058170

**Published:** 2013-03-05

**Authors:** Hongtuan Zhang, Hui Ma, Yong Xu, Liang Li

**Affiliations:** 1 Department of Urology, Second Affiliated Hospital of Tianjin Medical University, Tianjin Key Institute of Urology, Tianjin, China; 2 Department of Epidemiology, School of Public Health, Tianjin Medical University, Tianjin, China; 3 Laboratory of Population and Quantitative Genetics, School of Life Sciences, Tianjin Medical University, Tianjin, China; IPO, Inst Port Oncology, Portugal

## Abstract

**Background:**

Accumulating evidence has suggested that Mothers against decapentaplegic homolog 7 (SMAD7) rs12953717 polymorphism might be related to cancer risk. However, epidemiologic findings have been inconsistent. We therefore performed a meta-analysis to clarify the association between the SMAD7 rs12953717 polymorphism and cancer risk.

**Methods:**

A comprehensive search was conducted to identify all eligible studies of SMAD7 rs12953717 polymorphism and cancer risk. We used odds ratios (ORs) to assess the strength of the association, and 95% confidence intervals (CIs) to give a sense of the precision of the estimate. Heterogeneity, publication bias, and sensitivity analysis were also explored.

**Results:**

A total of 14 case-control studies, including 16928 cases and 14781 controls, were included in the present meta-analysis. The overall results showed that the variant genotypes were associated with a significantly increased risk of all cancer types (homozygote comparison, OR = 1.23, 95%CI = 1.10–1.38, P<0.01; heterozygote comparison, OR = 1.12, 95%CI = 1.02–1.22, P = 0.02; recessive model, OR = 1.17, 95%CI = 1.07–1.29, P<0.01; dominant model, OR = 1.15, 95%CI = 1.06–1.25, P<0.01; allelic model, OR = 1.12, 95%CI = 1.06–1.18, P<0.01). Further sensitivity analysis confirmed the significant association. In the subgroup analysis by ethnicity, SMAD7 rs12953717 polymorphism was significantly associated with cancer risk in both Caucasians and Asians. In the subgroup analysis by cancer types, SMAD7 rs12953717 polymorphism was significantly associated with colorectal cancer.

**Conclusions:**

Our investigations demonstrate that rs12953717 polymorphism is associated with the susceptibility of cancer. Large-scale and well-designed case-control studies are necessary to validate the risk identified in the present meta-analysis.

## Introduction

Cancer is the end of a complex disease that results from intricate interactions, including multi-factorial, multi-genetic and multi-stage processes [1]. It has become a worldwide public health burden [2], [3]. The complex etiology of this disease is not yet fully elucidated [4]. Recently, it has become clear that genetic variation contributes to the development and progression of cancer [4], [5]. However, due to various reasons, including considerable heterogeneity of cancer, the identification of susceptibility genes is difficult and most associations have not been replicated. Single nucleotide polymorphisms (SNPs) have attracted considerable attention in recent years as potential markers for predicting disease susceptibility and for guiding individualized therapeutic regimens. It is well known that the transforming growth factor-β (TGF-β) signaling pathway plays important roles in tumor initiation, invasion, and metastasis [6]. Genetic polymorphisms of genes that are involved in the TGF-β signaling pathways, including the mothers against decapentaplegic homolog 7 (SMAD7) gene, might impact susceptibility to cancer.

SMAD7 is an inhibitory SMAD and a negative regulator of the TGF-β signaling pathway that promotes the anti-inflammatory effects of TGF-β signaling via binding to TAB2 and TAB3 and inhibiting TAK1 [7]. It is known that a decrease in TGF-β signaling increases the risk for cancer. A variant in a component of this pathway may represent a suitable marker for identifying individuals at high risk of developing cancer [8]. Inactivating mutations of SMAD proteins have been found in human cancers [9]. Although SMAD7 protein overexpression has been shown to antagonize TGF-β-mediated fibrosis, carcinogenesis and inflammation, the underlying mechanism has not been fully elucidated [10]. Because SMAD7 is an inhibitory SMAD that acts as a negative regulator of the TGF-β signaling pathway, it is logical that genetic polymorphisms of SMAD7 might impact susceptibility to cancer. Several genetic variants within SMAD7, located on 18q21, have recently been investigated [11]–[22]. The rs12953717 polymorphism in intron 3 has been brought to our attention. A number of case-control studies have been conducted to investigate the association of rs12953717 with cancer susceptibility [11]–[20]. However, molecular epidemiological studies have yielded contradictory results concerning the potential role of SMAD7 rs12953717 polymorphism in cancer.

To date, no meta-analysis has been conducted to investigate the association of rs12953717 polymorphism of SMAD7 gene and cancer risk. Individual studies might have been underpowered to detect the overall effects. Some studies are limited by their sample size and subsequently suffer from too low power to detect effects that may exist. Given the amount of accumulated data, we deemed it important to perform a quantitative synthesis of the evidence. Hence, a meta-analysis based on a total of 14 independent studies was performed, which may provide the evidence for association of rs12953717 polymorphism with cancer susceptibility.

## Materials and Methods

### Literature Search

We searched for relevant publications using the terms “SMAD7”, “18q21”, “genetic susceptibility”, “SNP”, “inhibitory SMADs”, “polymorphism” or “variation”, “rs12953717” and “cancer” or “carcinoma” or “neoplasia” in PubMed, Cochrane Library and Embase electronic databases, and all eligible studies were published up to September 23, 2012. We evaluated all the retrieved publications to retrieve the most eligible literatures. Their reference lists were hand-searched to find other relevant publications. Of the studies with the same or overlapping data by the same investigators, we selected the most recent ones with the most subjects. As a prerequisite, only those published in English languages were included. Studies investigating more than one type of cancer with overlapping or same controls were regarded as individual data sets only in subgroup analyses by cancer type.

### Inclusion and Exclusion Criteria

The following inclusion criteria should be met: (1) evaluating the association between SMAD7 rs12953717 polymorphism and cancer risk, (2) using case–control design, (3) providing sufficient data for calculation of odds ratio (OR) with 95% confidence interval (CI), and (4) published in English languages. In addition, the following exclusion criteria were also used: (1) none-case-control studies; (2) no usable data reported; (3) the study only involved a case population; (4) animal studies; (5) pure cell studies; (6) not concerned with cancer risk; and (7) duplicated publications.

### Data Extraction

Two authors (Zahng H and Ma H) independently assessed the articles for compliance with the inclusion criteria. Disagreement was followed by discussion until consensus was reached. If these two authors could not reach a consensus, then a third author (Xu Y) was consulted to resolve the dispute. The following data were extracted: the name of the first author, publication year, and the Hardy-Weinberg equilibrium (HWE) among the controls, country of origin, cancer type, study type, smoking status, obesity status, source of controls, infection status, drinking status, genotyping methods, ethnicity of the population, and genotype distribution in cancer cases and controls. Different ethnicities were categorized as Caucasian, Asian and Mixed, which included more than one race. For case–control studies, data were extracted separately for each group whenever possible.

### Statistical Analysis

The strength of the association between SMAD7 rs12953717 polymorphism and cancer risk was measured by ORs, whereas a sense of the precision of the estimate was given by 95% CIs. We examined SMAD7 rs12953717 genotypes using the homozygote comparison (TT vs CC), heterozygote comparison (TC vs CC), recessive (TT vs TC+CC), dominant (TT+TC vs CC), and allelic (T vs C) models. Heterogeneity assumption was checked by Q-test. A significant Q-statistic (P<0.10) indicated heterogeneity across studies, and then the result of the random effect model was selected [23]. Otherwise, the result of the fixed effect model was selected [24]. To explore the reasons of heterogeneity, subgroup analyses were performed by ethnicity, study type, and cancer type.

The significance of the pooled OR was determined by the Z-test and P<0.05 was considered as statistically significant. The one-way sensitivity analyses were performed to assess the stability of the results, namely, a single study in the meta-analysis was deleted each time to reflect the influence of the individual data set to the pooled OR. An estimate of the potential publication bias was carried out by funnel plot. An asymmetric plot suggested a possible publication bias. The funnel plot asymmetry was assessed by Egger’s test. The significance of the intercept was determined by the t-test suggested by Egger. P<0.05 was considered representative of statistically significant publication bias. We assessed the departure from the HWE for the control group in each study using an online HWE calculator (http://ihg.gsf.de/cgi-bin/hw/hwa1.pl). All statistical tests were performed with the computer programs Review Manage 5.0 and Stata 11.0 using two-sided P-values.

## Results

### Characteristics of Studies

The process of selection of studies for inclusion in the meta-analysis is summarized in Figure S1. The database search identified 56 potentially relevant citations, of which 43 were judged to be of potential interest on the basis of the title. On the basis of the abstract, 27 studies were reviewed in their entirety. During the extraction of data,17 articles were excluded, because they did not provide sufficient data needed for OR calculation, were not for cancer research, were review articles or their contents associated with cancer prognosis and therapy, leaving 10 eligible articles including 14 studies [11]–[20]. The studies investigating different cancers, different ethnicity or different types of studies were separated into multiple studies in the subgroup analysis. Two of the eligible studies by the same authors used overlapping controls but targeted on different cancers, so we merged colorectal cancer (Broderick et al.-B) and chronic lymphocytic leukemia (Broderick et al.-2008) into one study in overall analysis and in subgroup analysis of Caucasians [19], [20]. Studies investigating more than one type of cancer with the same or overlapping controls were regarded as individual data sets in subgroup analyses by cancer type [13,19.20]. These 14 studies included 2 breast cancer studies [11], [17], 1 renal cancer study [16], 1 colorectal cancer, gastric cancer, and lung cancer study [13], 1 chronic lymphocytic leukemia study [19], and 11 colorectal cancer studies [12], [14], [15], [17], [18], [20]. Of these, there were 3 studies of Asians [12], [13], [16], 9 studies of Caucasians [14], [17]–[20], and 2 studies of mixed ethnicity [11], [15]. The genotype distributions in the controls of all studies were in agreement with HWE except for 2 studies [12], [15]. In addition, 13 studies [11]–[20] were replication-based and 1 study [20] was GAWS-based. The main characteristics for all eligible studies are listed in Table 1.

**Table 1:** Main characteristics of studies included in this meta-analysis.

Study	Year	Ethnicity	Cases			Controls			HWE	Study type	Cancer type	Genotyping method	Biological sample	Control source
			CC	TC	TT	CC	TC	TT						
Scollen et al.	2011	Mixed	710	1031	425	730	1083	437	Yes	Replication	Breast cancer	Taqman, SNPstream	Blood	PB
Ho et al.	2011	Asian	276	343	97	304	345	65	No	Replication	Colorectal cancer	Sequenom MassARRAY	Blood	HB
Li et al.	2011	Asian	154	241	11	90	63	13	Yes	Replication	Colorectal, gastric, lung cancer	Sequenom	Blood	PB
Slattery et al.	2010	Caucasian	503	754	332	676	928	327	Yes	Replication	Colon cancer	Illumina	Blood	PB
Hirata et al.	2009	Asian	125	68	17	139	48	13	No	Replication	Renal cancer	PCR-RFLP	Noncancerous kidney tissue, blood	HB
Gibson et al.	2009	Caucasian	375	630	244	277	431	179	Yes	Replication	Breast cancer	KASPar	Blood	PB
Thompson et al.	2009	Mixed	196	248	116	220	370	129	Yes	Replication	Colon cancer	Taqman	Blood	PB
Curtin et al. -Sheffield	2009	Caucasian	124	200	77	123	201	77	Yes	Replication	Colorectal cancer	SNPlex	-	PB
-Leeds		Caucasian	62	120	61	63	105	44	Yes	Replication	Colorectal cancer	SNPlex	-	PB
-Utah		Caucasian	128	210	88	146	215	67	Yes	Replication	Colorectal cancer	SNPlex	-	PB
Broderick et al.	2008	Caucasian	324	487	170	1250	1889	724	Yes	Replication	Chronic lymphocytic leukemia	KASPar	Blood	PB
Broderick et al.-A	2007	Caucasian	159	309	151	326	467	167	Yes	GWAS	Colorectal cancer	Illumina	Blood	PB
-B		Caucasian	1247	2204	973	1248	1898	722	Yes	Replication	Colorectal cancer	KASPar	Blood	PB
-C		Caucasian	582	991	422	558	834	312	Yes	Replication	Colorectal cancer	KASPar	Blood	PB
-D		Caucasian	277	468	198	106	168	67	Yes	Replication	Colorectal cancer	KASPar	Blood	PB

HWE, Hardy-Weinberg equilibrium; GWAS, genome-wide association studies; PCR-RFLP, polymerase chain reaction-restriction fragment length polymorphism; PB, population based; HB, hospital based.

### Meta-analysis

The detailed results of this meta-analysis, the publication bias test, and the heterogeneity test were presented in Tables 2. We first analyzed the association in the overall population. Then in order to obtain the exact consequence of the relationship between SMAD7 rs12953717 polymorphism and cancer susceptibility, stratified analyses by ethnicity, study type, and cancer type were performed. When the Q-test of heterogeneity was not significant, we conducted analyses using the fixed effect models. The random effect models were conducted when we detected significant between-study heterogeneity.

**Table 2:** Meta-Analysis of SMAD7 rs12953717 Polymorphism and Cancer.

Genetic model	Sample size		Egger’s test	Test of association		Analysis model	Heterogeneity
(No. of studies)	Case	Control	P value	OR (95% CI)	P		P value
**Overall(14)**							
TT vs CC	8624	7625	0.92	1.23 (1.10–1.38)	<0.01	R	<0.01
TC vs CC	13546	12162	0.38	1.12 (1.02–1.22)	0.02	R	<0.01
TT vs TC+CC	16928	14781	0.82	1.17 (1.07–1.29)	<0.01	R	0.01
TT+TC vs CC	16928	14781	0.39	1.15 (1.06–1.25)	<0.01	R	<0.01
T vs C	33856	29562	0.41	1.12 (1.06–1.18)	<0.01	R	0.01
**Study design**							
Replication study (13)							
TT vs CC	8314	7132		1.19 (1.08–1.32)	<0.01	R	0.06
TC vs CC	13078	11369		1.10 (1.00–1.21)	0.04	R	<0.01
TT vs TC+CC	16309	13821		1.15 (1.04–1.26)	<0.01	R	0.04
TT+TC vs CC	16309	13821		1.13 (1.04–1.22)	<0.01	R	0.01
T vs C	32618	27642		1.10 (1.05–1.15)	<0.01	R	0.09
**Cancer type**							
Colorectal cancer (11)							
TT vs CC	6132	5850		1.34 (1.24–1.44)	<0.01	F	0.17
TC vs CC	9537	9454		1.11 (1.02–1.22)	0.02	F	0.04
TT vs TC+CC	12058	11444		1.25 (1.17–1.33)	<0.01	F	0.38
TT+TC vs CC	12058	11444		1.17 (1.07–1.28)	<0.01	R	0.05
T vs C	24116	22888		1.15 (1.11–1.20)	<0.01	F	0.18
Breast cancer (2)							
TT vs CC	1754	1623		1.00 (0.87–1.15)	0.98	F	0.96
TC vs CC	2746	2312		1.01 (0.90–1.13)	0.87	F	0.42
TT vs TC+CC	3415	3137		1.00 (0.88–1.13)	0.94	F	0.69
TT+TC vs CC	3415	3137		1.01 (0.91–1.12)	0.89	F	0.53
T vs C	6830	6274		1.00 (0.93–1.07)	0.96	F	0.85
Others (3)							
TT vs CC	738	2229		0.90 (0.74–1.10)	0.30	F	0.11
TC vs CC	1263	3479		1.52 (0.86–2.66)	0.15	R	<0.01
TT vs TC+CC	1455	4229		0.73 (0.36–1.49)	0.39	R	0.02
TT+TC vs CC	1455	4229		1.41 (0.87–2.31)	0.17	R	<0.06
T vs C	2910	8458		1.17 (0.89–1.55)	0.27	R	0.02
**Ethnicity**							
Caucasian (9)							
TT vs CC	6497	5485		1.28 (1.19–1.38)	<0.01	F	0.11
TC vs CC	10154	8770		1.12 (1.06–1.19)	<0.01	F	0.90
TT vs TC+CC	12870	10732		1.19 (1.12–1.27)	<0.01	F	0.20
TT+TC vs CC	12870	10732		1.17 (1.10–1.23)	<0.01	F	0.52
T vs C	25740	21464		1.13 (1.09–1.17)	<0.01	F	0.13
Asian (3)							
TT vs CC	680	624		1.14 (0.58–2.23)	0.70	R	0.04
TC vs CC	1207	989		1.54 (0.97–2.42)	0.06	R	<0.01
TT vs TC+CC	1332	1080		0.92 (0.38–2.21)	0.84	R	<0.01
TT+TC vs CC	1332	1080		1.48 (1.08–2.03)	0.01	R	0.06
T vs C	2664	2160		1.25 (1.10–1.42)	<0.01	F	0.66
Mixed (2)							
TT vs CC	1447	1516		1.00 (0.86–1.16)	0.98	F	0.96
TC vs CC	2185	2403		0.88 (0.68–1.13)	0.31	R	0.07
TT vs TC+CC	2726	2969		1.05 (0.92–1.20)	0.46	F	0.31
TT+TC vs CC	2726	2969		0.95 (0.85–1.06)	0.32	F	0.17
T vs C	5452	5938		0.99 (0.92–1.07)	0.80	F	0.74

SMAD7, Mothers against decapentaplegic homolog 7; OR, odds ratio; CI, confidence interval.

#### Overall effects for meta-analysis

The association between SMAD7 rs12953717 and cancer risk was investigated in 14 studies with a total of 16928 cases and 14781 controls. Significant between-study heterogeneity was detected in all genetic models. In the overall analysis, we detected a significant association between SMAD7 rs12953717 polymorphism and cancer susceptibility under homozygote comparison (OR = 1.23, 95%CI = 1.10–1.38, P<0.01; Figure S2), heterozygote comparison (OR = 1.12, 95%CI = 1.02–1.22, P = 0.02; Figure S3), recessive model (OR = 1.17, 95%CI = 1.07–1.29, P<0.01; Figure S4), dominant model (OR = 1.15, 95%CI = 1.06–1.25, P<0.01; Figure S5), and allelic model (OR = 1.12, 95%CI = 1.06–1.18, P<0.01; Figure S6).

#### Subgroup analysis for study design

The overall results showed that the variant genotypes were associated with a significantly increased risk. After 1 GAWS-based study was excluded, the statistical significance of the results was not altered in replication studies. (homozygote comparison, OR = 1.19, 95%CI = 1.08–1.32, P<0.01; heterozygote comparison, R = 1.10, 95%CI = 1.00–1.21, P = 0.04; recessive model, OR = 1.15, 95%CI = 1.04–1.26, P<0.01; dominant model, OR = 1.13, 95%CI = 1.04–1.22, P<0.01; allelic model, OR = 1.10, 95%CI = 1.05–1.15, P<0.01).

#### Subgroup analysis for ethnicity

Subgroup analysis was stratified by ethnicity. The meta-analysis included 3 studies (1332 cases and 1080 controls) in Asian population, 2 studies(2726 cases and 2969 controls) in mixed population, and 9 studies (12870 cases and 10732 controls) in Caucasian population. In Caucasian population, the Q-test of heterogeneity was insignificant and we conducted analyses using fixed effect models in all genetic models. Statistically significant association was established for the SMAD7 rs12953717 polymorphism in Caucasians under all genetic models (homozygote comparison, OR = 1.28, 95%CI = 1.19–1.38, P<0.01; heterozygote comparison, R = 1.12, 95%CI = 1.06–1.19, P<0.01; recessive model, OR = 1.19, 95%CI = 1.12–1.27, P<0.01; dominant model, OR = 1.17, 95%CI = 1.10–1.23, P<0.01; allelic model, OR = 1.13, 95%CI = 1.09–1.17, P<0.01). For Asians, the heterogeneity was significant and we conducted analyses using random effect models except in the contrast of T versus C. The data suggested that rs12953717 was associated with cancer risk under dominant model (OR = 1.48, 95%CI = 1.08–2.03, P = 0.01) and allelic model (OR = 1.25, 95%CI = 1.10–1.42, P<0.01) in Asian population. When it comes to mixed population, we did not find any association between SMAD7 rs12953717 polymorphism and cancer susceptibility under all genetic models in mixed population.

#### Subgroup analysis for cancer types

Subgroup analysis was also stratified by cancer types. The meta-analysis included 11 studies (12058 cases and 11444 controls) based on colorectal cancer, 2 studies (3415 cases and 3137 controls) based on breast cancer, and 3 studies (1455 cases and 4229 controls) based on other cancers. In colorectal cancer subgroup, there was significant heterogeneity in the dominant model. There was no significant heterogeneity in the subgroup analysis of breast cancer. When it comes to other cancers, significant heterogeneity was found in all genetic models except in homozygote comparison. In different types of cancer, SMAD7 rs12953717 polymorphism was significantly associated with an increased risk of colorectal cancer in all genetic models (homozygote comparison, OR = 1.34, 95%CI = 1.24–1.44, P<0.01; heterozygote comparison, R = 1.11, 95%CI = 1.02–1.22, P = 0.02; recessive model, OR = 1.25, 95%CI = 1.17–1.33, P<0.00001; dominant model, OR = 1.17, 95%CI = 1.07–1.28, P<0.01; allelic model, OR = 1.15, 95%CI = 1.11–1.20, P<0.01). No evidence of association was found in any genetic model between SMAD7 rs12953717 polymorphism and the risk of breast cancer or other cancers.

### Sensitivity Analysis

In order to compare the difference and evaluate the sensitivity of the meta-analyses, we conducted one-way sensitivity analysis to evaluate the stability of the meta-analysis. The statistical significance of the results was not altered when any single study was omitted, confirming the stability of the results. Although the genotype distributions of control groups in 2 studies did not follow HWE, the corresponding pooled OR was not significantly altered by the exclusion of the 2 studies. Hence, results of the sensitivity analysis suggest that the data in this meta-analysis are relatively stable and credible. The detailed data were present in Table S1.

### Publication Bias

Begg’s funnel plot and Egger’s test were performed to assess the publication bias. The shape of funnel plots did not reveal any evidence of obvious asymmetry in all comparisons in overall population, and the Egger’s test was used to provide statistical evidence of funnel plot. The results did not show any evidence of publication bias in all comparisons. The detailed data were shown in Table 2.

## Discussion

Multiple lines of evidence supported an important role for genetics in determining risk for cancer, and association studies are appropriate for searching susceptibility genes involved in cancer [25]. In the recent years, interest in the genetic susceptibility to cancers has led to a growing attention to the study of polymorphisms of genes involved in tumourigenesis. Since the identification of SMAD7 rs12953717 polymorphism [20], an increasing number of studies suggested that SMAD7 rs12953717 polymorphism may play important roles in cancer development [11], [20]. Epidemiological studies of SMAD7 rs12953717 polymorphism, if large and unbiased, can provide insight into the *in vivo* relationship between the gene and cancer risk. However, studies focusing on the association of the SMAD7 rs12953717 polymorphism with cancer susceptibility had controversial conclusions. Some reviewed studies are limited by their sample size and subsequently suffer from too low power to detect effects that may exist. But the pool ORs generated from much larger population can increase the statistical power. Meta-analysis is a powerful tool for summarizing the results from different studies by producing a single estimate of the major effect with enhanced precision. It can overcome the problem of small sample size and inadequate statistical power of genetic studies of complex traits, and provide more reliable results than a single case-control study [26]. Combining data from many studies has the advantage of reducing random error [27].

In order to provide the comprehensive and reliable conclusion, we performed the present meta-analysis of 14 independent case–control studies. We investigated the association between the SMAD7 rs12953717 polymorphism and cancer risk. The subgroup analyses stratified by cancer types, ethnicity, and study type were also performed. To our knowledge, this study represented the first meta-analysis investigating the association between SMAD7 rs12953717 polymorphism and cancer risk. The results indicated that SMAD7 rs12953717 was significantly associated with cancer risk in overall analysis. These associations were very robust, which did not vary materially when the sensitivity analyses were performed. In the stratified analysis by cancer types, the results showed that SMAD7 rs12953717 polymorphism was significantly associated with increased colorectal risk. However, for breast cancer and other cancers, no associations were found in any genetic models. Results for different cancer types were inconsistent, which might be caused by the different microenvironments and mechanisms in different cancer types. Study results showed that lower median SMAD7 mRNA expression was associated with colorectal cancer risk allele at rs12953717 [20]. The SMAD7 acts as an intracellular antagonist of TGF-beta signaling by binding stably to the receptor complex and blocking activation of downstream signaling events [28]. Perturbation of SMAD7 expression has been previously documented to influence colorectal cancer progression [29], and loss of chromosome 18q21 encompassing SMAD7 is common in colorectal cancer [30]. The SMAD7 rs12953717 was identified in the GWAS for both adenomas and cancers [20]. As the number of copies of SMAD7 increases, the risk of colorectal cancer increases for carriers and the prognosis is worsened for patients with colorectal cancer [31]. SMAD7 expression is lower in cancers than adenomas irrespective of 18q copy number status makes a bystander effect unlikely and suggests a direct role for SMAD7 in carcinogenesis [32]. Hence, allele-specific expression of SMAD7 is likely to be the biological basis for colorectal cancer predisposition associated with 18q21 variation [32]. In the stratified analysis by ethnicity, we found that SMAD7 rs12953717 polymorphism was associated with increased cancer risk in both Caucasians and Asians.

Heterogeneity between studies is very common in the meta-analysis of genetic association studies. The between-study heterogeneity was also observed in our meta-analysis. Statistically significant between-study heterogeneity of genotype effect was detected in all genetic models when all the eligible studies were pooled into the meta-analysis. After subgroup analyses by ethnicity and cancer types, the heterogeneity was effectively removed or decreased. Therefore, it can be presumed that the relatively heterogeneity mainly results from differences of ethnicity and various cancer types. It is known that genotype distributions differ across populations, and genotype-phenotype associations may also depend on population stratification. In addition, there are some factors that could have contributed toward the heterogeneity. Definition of control group is different in different studies, the definition differences of the controls could have contributed to the heterogeneity observed in our meta-analysis. We attempted to determine if the heterogeneity might be explained by some variables such as smoking status, drinking status, and environmental factors included in the different studies, but are unable to provide a reliable answer to this question because we did not have access to individual level data for these variables. All above factors could have contributed to the between-study heterogeneity observed in our meta-analysis and the differences in the outcomes of the studies.

The current meta-analysis has several limitations which should be noted. Firstly, our search was restricted to studies published in indexed journals. This could bias the results of this review such as time-lag bias and publication bias. In time-lag bias [33], studies with ‘negative’ results take longer time to be published, whereas enthusiastic results are published much more quickly. In publication bias [34], [35], small studies with ‘negative’ results are never published, whereas equally small studies with similar quality but ‘positive’ results would appear in the literature. Secondly, the controls were not uniform. Non-differential misclassification bias was possible because these studies may have included controls that are likely to develop cancer in subsequent years though they had no clinical symptoms at the time of investigation. Thirdly, the results were based on unadjusted ORs, while a more precise estimation should take into account the effect of multiple confounders such as age, smoking status, drinking status and environmental factors on the association. Lack of information for data analysis may cause serious confounding bias. Forthly, our analysis was limited to Asian, Caucasian, and mixed ethnicities, so it is uncertain whether these results are generalizable to other populations. For Asians and mixed population, the number of the included studies was limited and their sample sizes were small. It may be underpowered to explore the real association. Thus, the results should be interpreted with care. In addition, our analysis did not consider the possibility of gene-gene or SNP-SNP interactions or the possibility of linkage disequilibrium between polymorphisms. Further investigations of the haplotypic effect of a gene and the study of multiple polymorphisms in different genes are needed.

### Conclusions

In conclusion, our meta-analysis revealed that rs12953717 polymorphism of SMAD7 involved in the TGF-βsignaling pathway was significantly associated with cancer susceptibility. Due to limitations showed above in this analysis, it is critical that larger and well-designed studies are needed to confirm our results.

## Supporting Information

Figure S1 **The flow diagram for the review process and outcomes of inclusion and exclusion.**
(DOC)

Figure S2 **Forest plot of ORs with 95% CI for SMAD7 rs12953717 polymorphism and overall cancer risk (TT versus CC).**
(PNG)

Figure S3 **Forest plot of ORs with 95% CI for SMAD7 rs12953717 polymorphism and overall cancer risk (TC versus CC).**
(PNG)

Figure S4 **Forest plot of ORs with 95% CI for SMAD7 rs12953717 polymorphism and overall cancer risk (TT versus TC + CC).**
(PNG)

Figure S5 **Forest plot of ORs with 95% CI for SMAD7 rs12953717 polymorphism and overall cancer risk (TT + TC versus CC).**
(PNG)

Figure S6 **Forest plot of ORs with 95% CI for SMAD7 rs12953717 polymorphism and overall cancer risk (T versus C).**
(PNG)

Table S1 ORs (95% CI) of sensitivity analysis for SMAD7 rs12953717.
(DOC)
