# Supplementary material for: Association of SMAD7 rs12953717 Polymorphism with Cancer: A Meta-Analysis
Source: PLoS One. 2013 Mar 5;8(3):e58170. doi: 10.1371/journal.pone.0058170 (PMC3589366; doi:10.1371/journal.pone.0058170)
Supplement: Figure S1 — The flow diagram for the review process and outcomes of inclusion and exclusion. (DOC) [file pone.0058170.s001.doc]

Manuscripts were excluded: Improper titles (n=13)

Potentially relevant manuscripts retrieved for abstract evaluation (n=43)

Manuscripts excluded after abstract review (n=16)

Potentially relevant manuscripts retrieved for detailed review (n=27)

10 eligible articles including 14 independent studies

17 studies were excluded due to:

Articles did not explore cancer risk (n=14)

Review articles (n=1)

Insufficient data for calculation of OR and 95%CI (n=2)

Potentially relevant manuscripts were searched from Pubmed, Cochrane Library and Embase electronic databases up to September 23, 2012 (n=56)
