# Supplementary figures and images for: Association of SMAD7 rs12953717 Polymorphism with Cancer: A Meta-Analysis
Source: PLoS One. 2013 Mar 5;8(3):e58170. doi: 10.1371/journal.pone.0058170 (PMC3589366; doi:10.1371/journal.pone.0058170)

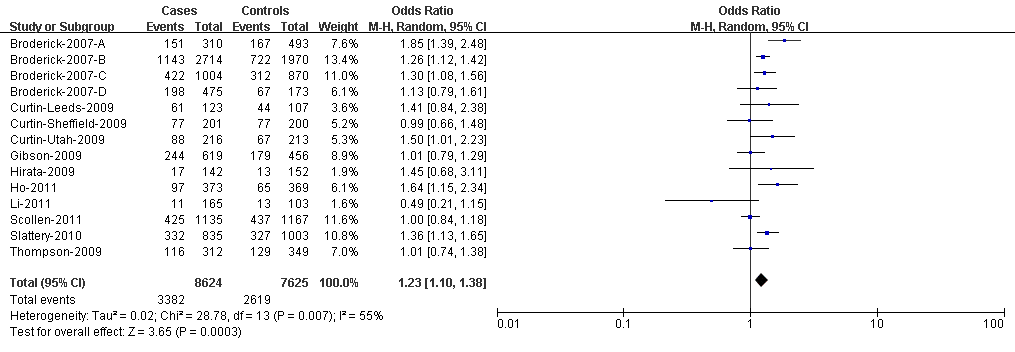

Supplement: Figure S2 — Forest plot of ORs with 95% CI for SMAD7 rs12953717 polymorphism and overall cancer risk (TT versus CC). (PNG) [file pone.0058170.s002.png]

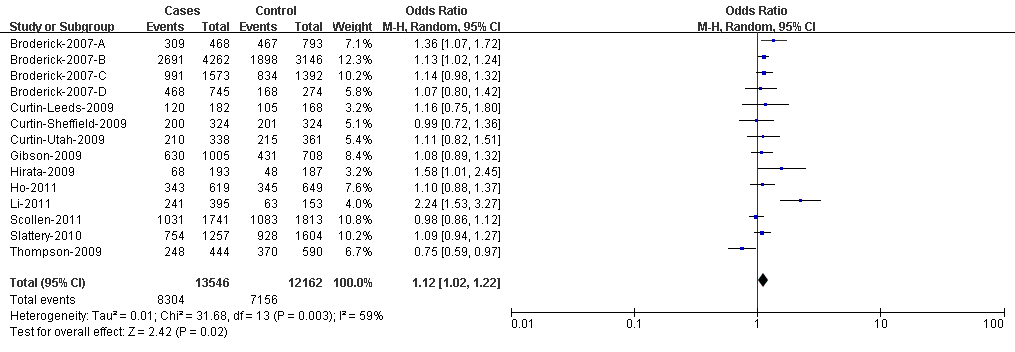

Supplement: Figure S3 — Forest plot of ORs with 95% CI for SMAD7 rs12953717 polymorphism and overall cancer risk (TC versus CC). (PNG) [file pone.0058170.s003.png]

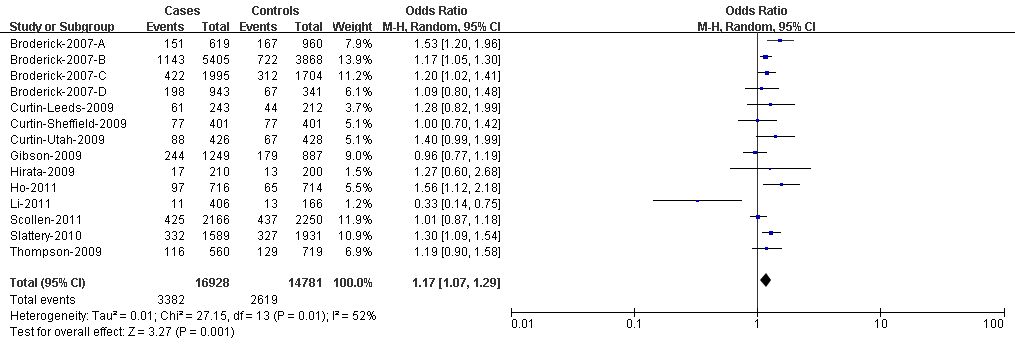

Supplement: Figure S4 — Forest plot of ORs with 95% CI for SMAD7 rs12953717 polymorphism and overall cancer risk (TT versus TC + CC). (PNG) [file pone.0058170.s004.png]

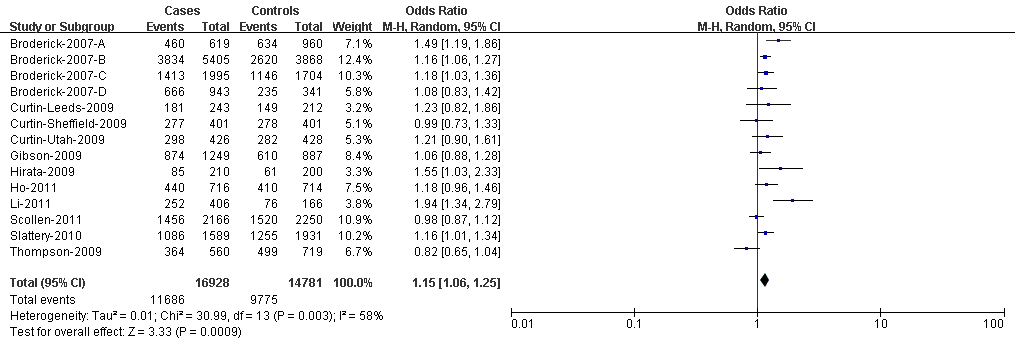

Supplement: Figure S5 — Forest plot of ORs with 95% CI for SMAD7 rs12953717 polymorphism and overall cancer risk (TT + TC versus CC). (PNG) [file pone.0058170.s005.png]

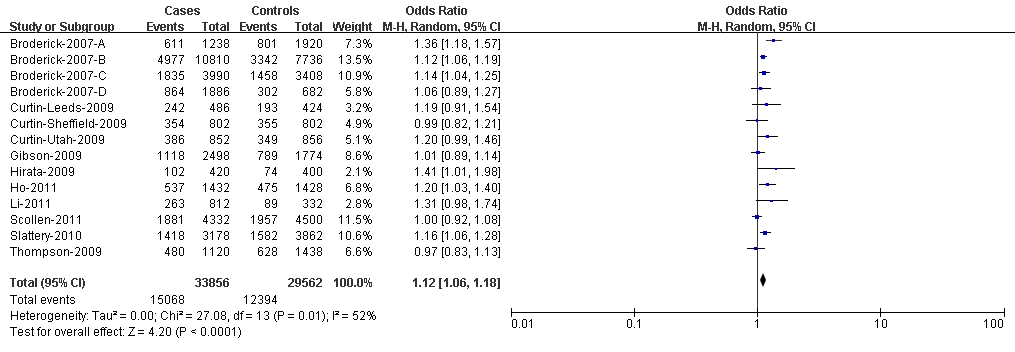

Supplement: Figure S6 — Forest plot of ORs with 95% CI for SMAD7 rs12953717 polymorphism and overall cancer risk (T versus C). (PNG) [file pone.0058170.s006.png]
