# Supplementary material for: Association of SMAD7 rs12953717 Polymorphism with Cancer: A Meta-Analysis
Source: PLoS One. 2013 Mar 5;8(3):e58170. doi: 10.1371/journal.pone.0058170 (PMC3589366; doi:10.1371/journal.pone.0058170)
Supplement: Table S1 — ORs (95% CI) of sensitivity analysis for SMAD7 rs12953717. (DOC) [file pone.0058170.s007.doc]

**Table S1. ORs (95% CI) of sensitivity analysis for SMAD7 rs12953717**

| Excluding literature | TT vs CC | TC vs CC | TT vs TC + CC | TT + TC vs CC | T vs C |
| --- | --- | --- | --- | --- | --- |
| one by one | OR (95% CI) | OR (95% CI) | OR (95% CI) | OR (95% CI) | OR (95% CI) |
| Total | 1.23 (1.10-1.38) | 1.12 (1.02-1.22) | 1.17 (1.07-1.29) | 1.15 (1.06-1.25) | 1.12 (1.06-1.18) |
| Broderick-2007-A | 1.19 (1.08-1.32) | 1.10 (1.00-1.21) | 1.15 (1.04-1.26) | 1.13 (1.04-1.22) | 1.10 (1.05-1.15) |
| Broderick-2007-B | 1.23 (1.07-1.40) | 1.12 (1.01-1.25) | 1.17 (1.05-1.32) | 1.16 (1.05-1.28) | 1.12 (1.05-1.19) |
| Broderick-2007-C | 1.22 (1.08-1.39) | 1.12 (1.01-1.23) | 1.17 (1.05-1.30) | 1.15 (1.05-1.26) | 1.12 (1.05-1.19) |
| Broderick-2007-D | 1.24 (1.10-1.39) | 1.12 (1.02-1.23) | 1.18 (1.07-1.31) | 1.16 (1.06-1.27) | 1.12 (1.06-1.19) |
| Curtin-2009-Leeds | 1.23 (1.09-1.38) | 1.12 (1.02-1.23) | 1.17 (1.06-1.29) | 1.15 (1.06-1.25) | 1.12 (1.06-1.18) |
| Curtin-2009-Sheffield | 1.25 (1.11-1.40) | 1.13 (1.02-1.24) | 1.18 (1.07-1.31) | 1.16 (1.07-1.27) | 1.13 (1.07-1.19) |
| Curtin-2009-Utah | 1.22 (1.08-1.37) | 1.12 (1.02-1.23) | 1.16 (1.05-1.28) | 1.15 (1.05-1.26) | 1.12 (1.06-1.18) |
| Gibson-2009 | 1.26 (1.12-1.41) | 1.12 (1.02-1.24) | 1.20 (1.08-1.32) | 1.16 (1.06-1.27) | 1.13 (1.07-1.19) |
| Hirata-2009 | 1.23 (1.09-1.38) | 1.10 (1.01-1.21) | 1.17 (1.06-1.29) | 1.14 (1.05-1.24) | 1.11 (1.06-1.17) |
| Ho-2011 | 1.21 (1.08-1.35) | 1.12 (1.02-1.23) | 1.15 (1.05-1.27) | 1.15 (1.05-1.26) | 1.11 (1.05-1.18) |
| Li-2011 | 1.25 (1.12-1.39) | 1.09 (1.03-1.14) | 1.17 (1.11-1.24) | 1.13 (1.05-1.21) | 1.11 (1.06-1.18) |
| Scollen-2011 | 1.27 (1.13-1.42) | 1.14 (1.03-1.25) | 1.20 (1.08-1.32) | 1.17 (1.08-1.28) | 1.13 (1.08-1.19) |
| Slattery-2010 | 1.22 (1.08-1.38) | 1.12 (1.02-1.24) | 1.16 (1.04-1.29) | 1.15 (1.05-1.27) | 1.12 (1.05-1.18) |
| Thompson-2009 | 1.25 (1.11-1.41) | 1.14 (1.05-1.24) | 1.17 (1.06-1.30) | 1.18 (1.09-1.27) | 1.13 (1.07-1.19) |
| Studies did not follow the HWE | 1.20 (1.07-1.35) | 1.11 (1.00-1.22) | 1.15 (1.04-1.27) | 1.14 (1.04-1.25) | 1.11 (1.05-1.17) |

SMAD7, Mothers against decapentaplegic homolog 7; OR, odds ratio; CI, confidence interval
